# Nitrogen Minimization in Hydrothermal Liquefaction Biocrude from Sewage Sludge with Green Extraction Solvents

**DOI:** 10.1021/acsomega.4c00455

**Published:** 2024-03-15

**Authors:** Muhammad Usman, Shuo Cheng, Sasipa Boonyubol, Jeffrey S. Cross

**Affiliations:** Department of Transdisciplinary Science and Engineering, School of Environment and Society, Tokyo Institute of Technology, 2-12-1, Ookayama, Meguro-ku, Tokyo 152-8550, Japan

## Abstract

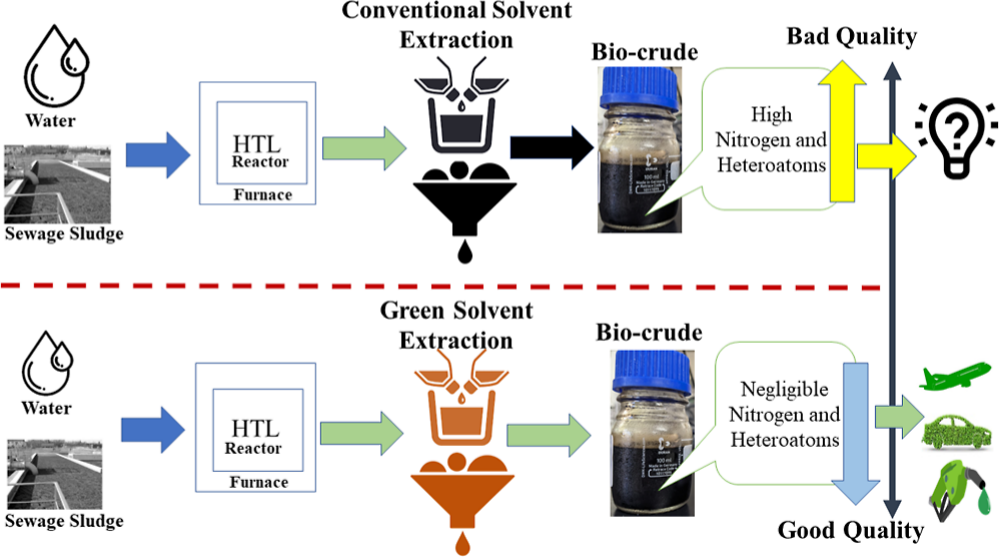

This study explored the effectiveness of hydrothermal liquefaction (HTL) in converting sewage sludge (SS) into high-quality biocrude. It scrutinized the influence of various solvents, including conventional choices like dichloromethane (DCM) and hexane, alongside environmentally friendly alternatives, such as ethyl butyrate (EB) and ethyl acetate (EA). HTL experiments, conducted at 350 °C for 60 min in a 20 mL batch reactor, include solvent-based biocrude extraction. Notably, EB showed the highest extraction yield (50.1 wt %), the lowest nitrogen distribution (5.4% with 0.32 wt %), and a remarkable 74% energy recovery (ER), setting a noteworthy benchmark in nitrogen reduction. GCMS analysis reveals EB-derived biocrude’s superiority in having the least heteroatoms and nitrogenous compounds compared to hexane, EA, and DCM. Solid residues from hexane, EB, and EA displayed the highest nitrogen distribution range (62–68%), hinting at potential applications in further processes. These findings significantly inform solvent selection for efficient and sustainable waste-to-energy conversion. While promising, the study emphasizes the need to explore solvent–solute interactions further to optimize biocrude quality, highlighting the pivotal role of solvent choice in advancing clean, cost-effective waste-to-energy technologies.

## 1 Introduction

The increasing urban population and enhanced sewage treatment have led to a substantial rise in sewage sludge (SS) production globally. Notably, China annually produces about 20 million metric tons (Mt) (dry matter) of SS, Europe around 10 Mt, Japan contributes 2.2 Mt, and the United States manages 49 trillion liters each year.^1−4^ This growing volume of SS, a complex mix of organic and inorganic materials, poses environmental risks and disposal challenges,^4,5^ necessitating innovative and sustainable management strategies in line with urban expansion.

Hydrothermal liquefaction (HTL) stands as a progressive technology in waste management and sustainable energy. It operates under moderate conditions, typically 250–350 °C and 5–25 MPa, distinguishing itself by converting various feedstocks, including wet and dry SS, into biocrude, organically rich aqueous phase, solid biochar, and gases.^6^ This technology offers considerable benefits, like waste volume reduction, lower greenhouse gas emissions, and resource extraction from SS.^5−7^ However, challenges arise in managing heteroatoms [e.g., heterocyclic, oxygen (O), and sulfur (S)] and nitrogen (N) compounds in HTL products, particularly biocrude derived from protein-rich SS, necessitating innovative approaches to optimizing its use as biofuel.^8−10^

Efforts to improve biocrude’s commercial viability as a fuel have involved catalytic cracking, hydrotreating, emulsification, and blending.^9,10^ However, biocrude’s high N content poses a significant challenge. During combustion, this N can lead to environmental pollution through nitrogen oxide (NOx) emissions.^11,12^ In the HTL process, about 20–40% of biomass nitrogen transfers to the biocrude, resulting in N concentrations as high as 10 wt %,^13^ which is much higher than the 0.1–1.5 wt % typically found in petroleum crude.^14^ This elevated N level in biocrude underscores a critical hurdle in its development as a sustainable fuel.

In batch HTL systems, the biocrude is separated from other byproducts using extraction solvents like acetone,^15^ dichloromethane (DCM),^16^ toluene,^17^ and methanol.^18^ These solvents are pivotal for maximizing biocrude yields and efficient byproduct separation. Recent studies have highlighted their impacts on biocrude quality and energy conversion. One study found DCM to yield the highest biocrude percentage at 48.8% across different algae strains.^19^ Another study compared DCM, hexane, toluene, and acetone for extracting biocrude from municipal SS, yielding 38, 12, 18, and 10%, respectively, on a dry-ash-free basis. Notably, the N content in the biocrude varied significantly among these solvents, ranging from 4.2 to 5.7%.^20^ These findings emphasize that solvent choice not only affects biocrude yield but also the N content in the resultant biocrude, making the selection critical for the process’s efficiency and economic feasibility.

Green solvents are an alternative to conventional organic solvents for the production of bio-oil because of their safety, stability, recyclability, and biodegradability. The exploration and adoption of green solvents have garnered substantial attention across diverse industries, including pharmaceuticals, chemicals, and biofuels,^21^ driven by the pursuit of sustainability and environmental responsibility. However, these eco-friendly solvents have not been previously examined or reported in the extraction of biocrude, particularly from HTL of SS. Therefore, this study aims to investigate the impact of green solvents on SS HTL biocrude quality, focusing on heteroatoms and N-contents, and compare the results with those obtained using conventional organic solvents, addressing a notable research gap in the field.

## 2 Material and Methods

### 2.1 Sample Collection

Dewatered SS samples, which contain ∼75 to 80% water contents^4^ and are produced from the dewatering step (belt or screw press) before drying, were collected from the wastewater treatment facility in Tokyo, Japan.

### 2.2 Chemicals

Primary solvents (DCM, hexane, EA (ethyl acetate), and EB (ethyl butyrate) from Sigma-Aldrich, US) extracted biocrude from HTL products. DCM and hexane, commonly used, are effective for bio-oil extraction. This study introduces green solvents (EA and EB) known for their eco-friendly attributes, aiming to assess their impact on HTL product quality compared to traditional solvents.

### 2.3 HTL Experiment

This study utilized 20 mL mini-autoclave reactors for HTL experiments, and the reactor assembly, including 1/2 in. 316 stainless steel part connectors, caps, and screws, was sourced from the Japanese vendor Monotaro (Osaka soul, Tokyo, Japan). Cost-effective expanded graphite gaskets, known for their high temperature resistance, ensured effective sealing. Operating at 350 °C for 60 min with a sludge-to-water loading ratio of 1:9, all experiments were conducted in duplicate for reliability. These conditions are recognized for achieving optimal biocrude yields, or SS conversion.^20,22^ Reactors were loaded with SS and water and heated at approximately 10 °C/min until reaching the required temperature.

### 2.4 HTL Products Separation

**Figure 1:**
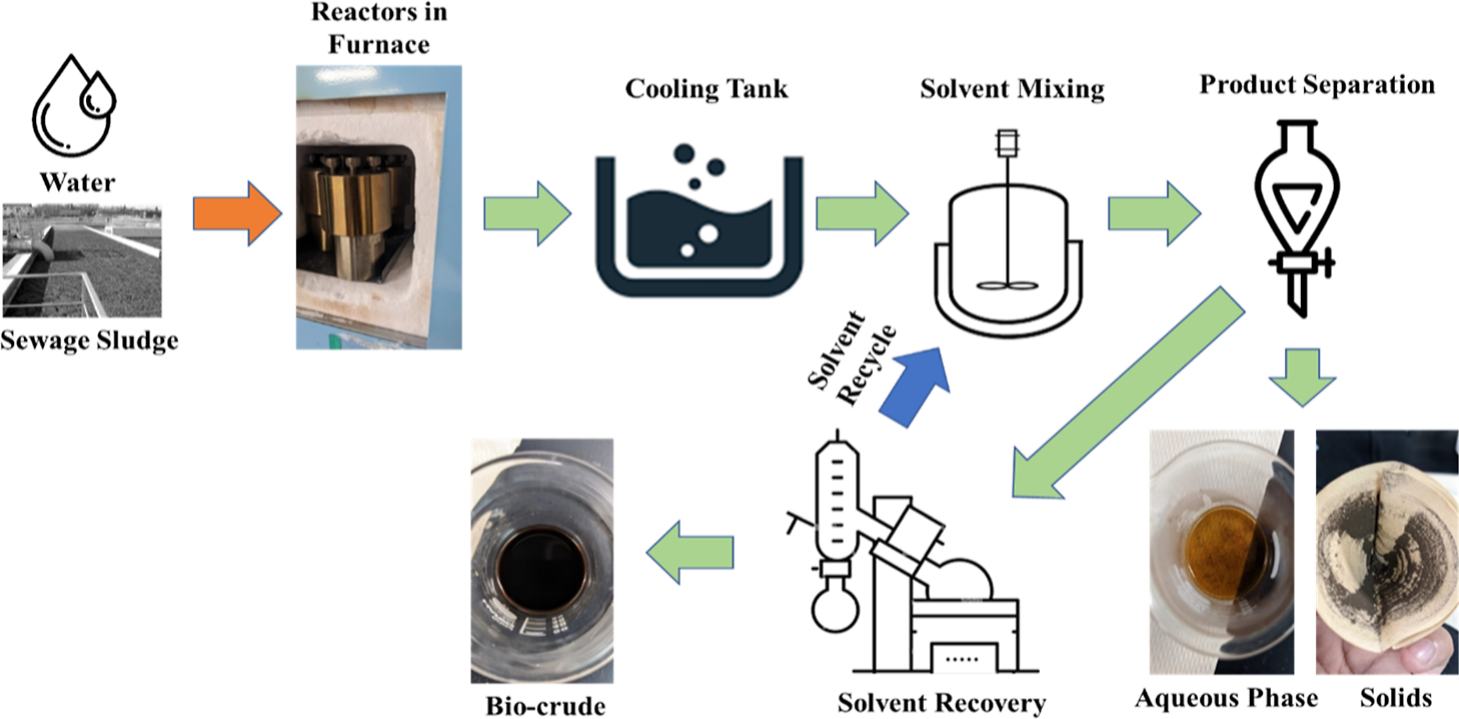
HTL of SS experimental flow and product separation.

After the designated reaction period, reactors were swiftly cooled in an ambient-temperature water bath and dried for 30 min, ensuring exterior dryness and internal equilibration to room temperature. Upon depressurizing and opening, liquid and solid products were recovered and mixed with 20 mL of extraction solvents for 30 min on a magnetic stirring plate. Filtration through a 110 mm JIS P 3801 Class 1 filter paper separated the solid residue and dried at 105 °C for 24 h. The biocrude and aqueous phase layers were split into a separating funnel. The aqueous phase underwent drying at 80 °C for the dissolved organics. Solvent-extracted biocrude was obtained by using a rotary evaporator for DCM, hexane, and EA, and a Soxhlet extractor for EB. Solvents were largely recycled with a recovery rate of approximately 95%, acknowledging minor losses during condensation and valve operations. The experimental workflow, including HTL of SS and product separation, is detailed in Figure 1.

Throughout the experiment, all product weights were meticulously recorded for a comprehensive mass balance. Product yields (on a dry-ash-free basis) were calculated using predefined formulas (eq 1), and the gas yield was determined by difference as per eq 2.
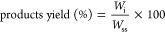
1
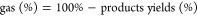
2where *W* is weight and *i* represents biocrude, aqueous phase, or solids.

### 2.5 Characterization Techniques

To conduct a thorough analysis, SS samples and HTL products underwent elemental (CHNS) analysis using an organic elemental analyzer (JM10, J-Science Lab Co. Ltd., Japan). The determination of the oxygen content relied on the calculation of differences. To assess the higher heating value (HHV) of both SS samples and HTL biocrudes, the Dulong formula (eq 3) was applied. This formula incorporates the weight percentages (wt %) of elements C, H, O, and S in both the SS and biocrude samples.
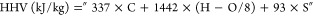
3

Energy recovery (ER) in each product was calculated using eq 4.
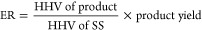
4

In addition, nitrogen distribution (ND) is characterized by the quantity of nitrogen present in the product in relation to the nitrogen content in SS. This calculation is based on eq 5, utilizing the CHNS results for each HTL products.
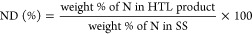
5

Biocrude composition was assessed using a Shimadzu QP 2010 GC–MS instrument equipped with an Rxi-5Sil MS column (30 m length, 0.25 mm inner diameter, and 0.25 μm film thickness). An autosampler injected 1 μL of the sample at an inlet temperature of 230 °C for swift vaporization. Helium (He) served as the carrier gas at a flow rate of 1.5 mL/min. The temperature sequence included an initial 60 °C for 5 min, followed by increments to 120 °C at 2.5 °C/min, 240 °C at 5 °C/min, and 320 °C at 5 °C/min, with a 5 min hold. In this study, precise characterization was achieved by utilizing peak retention times and areas in the GC–MS analysis. This approach facilitated the assessment of compound identification, molecular weight determination, and chemical composition analysis of the samples.

## 3 Results and Discussion

### 3.1 HTL Products

**Figure 2:**
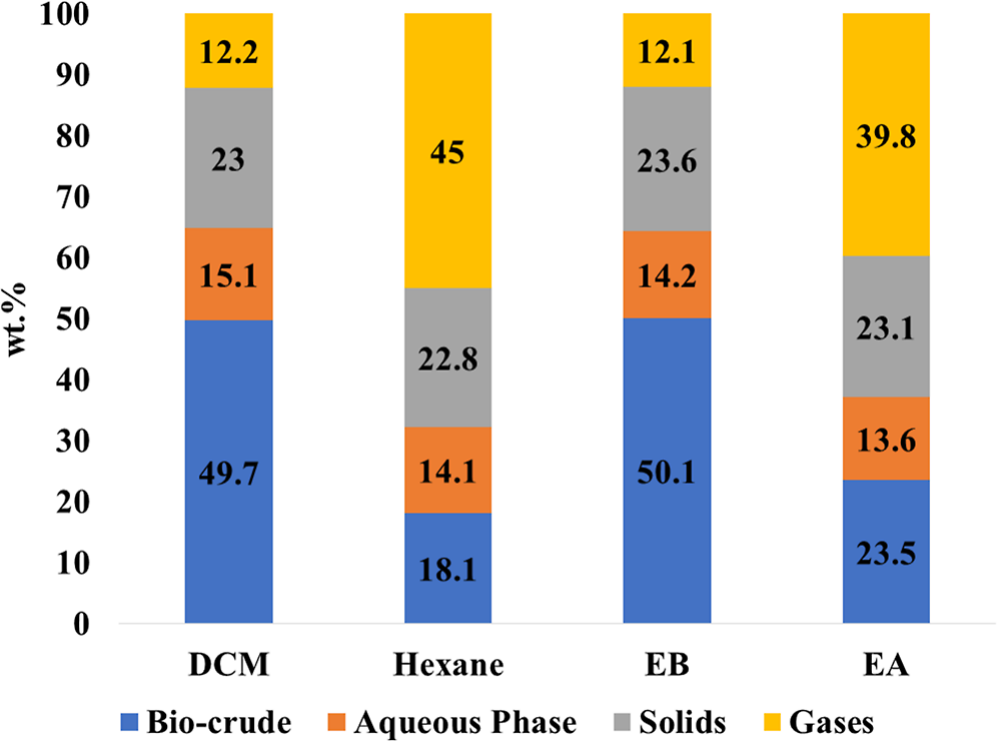
Product yield of the SS HTL experiment at 350 °C.

Figure 2 demonstrates the distribution of products from the HTL of sewage sludge at 350 °C, resulting in biocrude, aqueous phase, solids, and gases. These products collectively represent about 100% of the SS feedstock on a dry basis, with a potential error margin of ±5% due to factors such as volatile evaporation during solvent separation and experimental inaccuracies.

DCM exhibited superior biocrude extraction among the organic solvents, achieving a notable extraction yield of 49.7 wt %, consistent with findings reported in the literature that consistently highlight higher yields with DCM.^19,20,22,23^ This is attributed to DCM’s efficiency, which stems from the fact that it can efficiently extract fatty acids, ester derivatives, and cyclic and noncyclic oxygenates.^24^ Hexane gave a significantly lower biocrude extraction yield (18.1 wt %) compared to DCM, which is also similar to the literature.^20^ Several factors could contribute to this observation. Hexane and DCM have different solvating properties due to variations in polarity and chemical structure. Hexane, being a nonpolar solvent, may have a lower affinity for certain polar compounds present in the biocrude. In contrast, DCM, being a polar aprotic solvent, might be more effective in dissolving a broader range of compounds.

On the other hand, EB also showed higher biocrude extraction yields of 50.1 wt %, which is comparable to DCM and EA, which showed 23.5 wt %. The variation in extraction yields among the solvents can be attributed to their different solvating powers. These solvents, hexane, EB, and EA (slightly polar), share a common trait of being nonpolar. They are recognized for their ability to selectively extract nonpolar hydrocarbons.^4,25−27^ It has been reported that SS contains a diverse range of both polar and nonpolar chemical compounds. The predominant composition of sludge consists of nonpolar compounds,^28^ aligning with the extraction yields observed with EB due to its nonpolar nature.

However, the other products, namely the aqueous phase and solids, remained consistent across all four extraction solvents as they were produced under the same HTL conditions. Nevertheless, the yields of gases were notably higher in hexane and EA, attributed to the lower polar extraction nature of these solvents. This phenomenon suggests that the remaining biocrude may have mixed with the solids during the extraction process.

In conclusion, these results underscore the promising potential of using green solvents as alternatives to traditional organic solvents, showcasing comparable extraction yields. However, it is crucial to highlight that the primary focus of this study is on the heteroatoms and N-containing compounds present in the HTL products, particularly in biocrudes. Analyzing the elemental composition of these biocrudes becomes imperative to assess and ensure their quality, especially concerning N-contents, providing a more comprehensive understanding of the effectiveness of green solvents in preserving biocrude quality during the HTL.

### 3.2 Elemental Composition of HTL Biocrudes

Table 1 presents the elemental compositions and HHVs of both raw SS and biocrudes acquired from HTL of SS at 350 °C. The overall findings indicate favorable quality in terms of carbon (C), hydrogen (H), N, and S for all four extracted biocrudes compared to sewage sludge. Notably, hexane stands out as the leader in quality and HHV, followed by EB, EA, and DCM, as determined from the CHNS results.

**Table 1:** Elemental Compositions of SS and HTL Biocrude^a^

	elements					
	C (wt %)	H (wt %)	N (wt %)	S (wt %)	O (wt %)	HHV (MJ/kg)
SS	44.34	6.37	5.89	0.62	31.48	18.52
biocrude extracted with						
DCM	54.9	6.9	3.96	0.53	33.62	22.44
hexane	61.62	12.3	0.23	<LOD	25.85	33.84
EB	58.87	9.18	0.32	0.03	31.6	27.38
EA	47.3	8.47	0.23	<LOD	43.96	20.23

a “Oxygen composition calculation equation”—O % = 100% – C % – H % – N % – S %. LOD—Limit of detection.

An important observation lies in the alteration of N contents and other heteroatoms like S and O (calculated by difference) in the biocrudes derived from SS. DCM exhibited the highest extraction of N contents at 3.96 and 0.53 wt % of S, posing challenges and indicating lower quality compared to others. In contrast, hexane, EB, and EA showed nearly negligible extractions, with 0.23, 0.32, and 0.23 wt % of N contents, respectively, and S contents were below detection limits. It is noteworthy that hexane, EB, and EA are nonpolar solvents, while DCM is polar, and N and S compounds often exhibit polar characteristics. DCM’s polarity allows it to interact more favorably with these compounds, leading to better extraction efficiency.^29,30^ In addition to this, DCM can undergo nucleophilic substitution (SN2) reactions and has a higher affinity for polar and nonpolar compounds. This versatility enables DCM to efficiently extract a wide range of substances, including N and S compounds.^31,32^ It has been reported that DCM is utilized to extract N and S compounds from aqueous solutions in sequential extraction processes after HTL of SS.^33^

On the other hand, nonpolar solvents are suitable for certain extractions, but they may not effectively interact with polar N and S compounds. These findings lead to the conclusion that nonpolar solvents, exemplified by EB, which demonstrated high extraction yields with fewer heteroatoms, are better suited for biocrude extraction from HTL of SS. This suitability arises from the composition of SS, which includes proteins, lipids, and carbohydrates as the primary sources of heteroatoms.^23^ However, it is crucial to identify the chemical compounds in the extraction for their valorization and comprehensive understanding.

### 3.3 Chemical Compounds in Biocrudes

**Figure 3:**
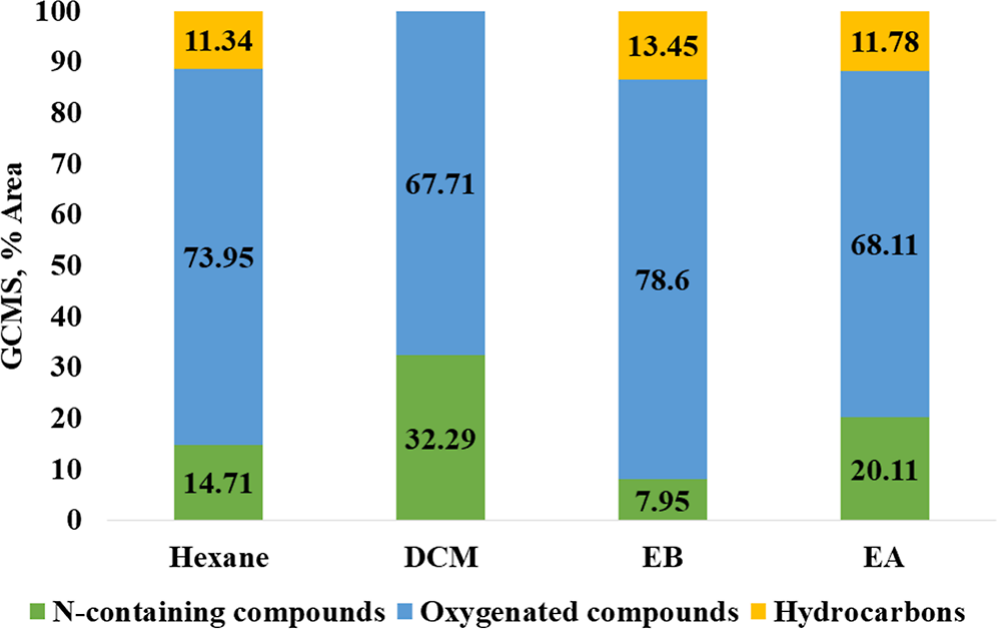
Distribution of chemical compounds in extracted biocrudes identified by GCMS.

The biocrude, as characterized by GCMS results, is categorized into distinct groups: “N-containing compounds (N&O heterocyclic compounds and amides), oxygenated compounds (ketones, aldehydes, alcohols, acids, and esters), and hydrocarbons”, as depicted in Figure 3.

All biocrudes were found to be predominantly made up of oxygenated compounds, followed by N-containing compounds and hydrocarbons. These findings align with a previous study result.^34^ The abundance of oxygenated compounds in SS HTL biocrude can be attributed to the complex composition of SS, which contains various organic materials such as lipids, proteins, and carbohydrates.^4^ However, a notable observation is that the N-containing compounds in EB are significantly lower (7.95%), followed by hexane (14.71%), EA (20.11%), and DCM (32.29%) based on GCMS percentage area, which is attributed to their polar and nonpolar nature. The analysis was repeated for several experiments to ensure the reliability and consistency of the results.

**Figure 4:**
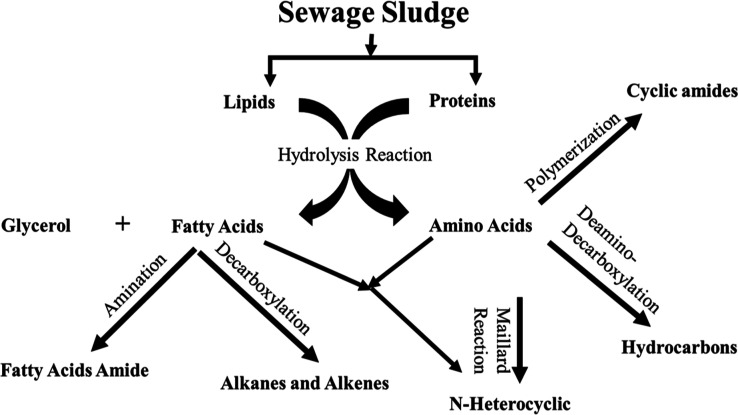
Possible reaction pathways of these chemical compounds in extracted biocrudes.

The comprehensive details of chemical compounds identified using GCMS, such as percentage area and chemical compounds, are available in Table 2. Elaborating on the reaction pathways of SS in HTL and the chemical compounds in biocrude proves challenging due to the complex nature of SS. Nevertheless, Figure 4 outlines potential reactions that could occur in the HTL of SS and the formation of these compounds.

**Table 2:** Chemical Compounds and Related Percentage Area Identified by GCMS in All Extracted Biocrudes

sr no.	DCM chemical compounds	% area	hexane chemical compounds	% area	EB chemical compounds	% area	EA chemical compounds	% area
1	2-cyclopenten-1-one, 2-methyl-	5.23	2-cyclopenten-1-one, 2-methyl-	3.23	butanoic acid, propyl ester	2.08	2-cyclopenten-1-one, 2-methyl-	4.07
2	pyrazine, 2,6-dimethyl-	3.85	pyrazine, ethyl-	2.42	2-cyclopenten-1-one, 2-methyl-	3.36	pyrazine, 2,6-dimethyl-	2.71
3	pyrazine, ethyl-	6.49	pyrazine, 2-ethyl-6-methyl-	2.13	pyrazine, 2,6-dimethyl-	2.25	pyrazine, ethyl-	3.95
4	2-cyclopenten-1-one, 3-methyl-	2.34	pyrazine, 2-ethyl-3-methyl-	3.93	pyrazine, ethyl-	3.38	2-cyclopenten-1-one, 3-methyl-	1.83
5	pyrazine, 2-ethyl-6-methyl-	1.75	benzene, 1-ethoxy-4-methyl-	1.87	2-cyclopenten-1-one, 3-methyl-	1.96	phenol	1.67
6	pyrazine, 2-ethyl-3-methyl-	3.49	cyclohexanone, 3-ethenyl-	2.38	phenol	5.23	pyrazine, 2-ethyl-6-methyl-	1.89
7	2-cyclopenten-1-one, 2,3-dimethyl-	2.57	diethyl phthalate	2.82	pyrazine, 2-ethyl-3-methyl-	2.93	pyrazine, 2-ethyl-3-methyl-	3.5
8	benzene, 1-ethoxy-4-methyl-	1.76	1-hexadecanol	4.04	2-cyclopenten-1-one, 2,3-dimethyl-	2.94	2-cyclopenten-1-one, 2,3-dimethyl-	2.1
9	1-hexadecanol	3.17	*n*-hexadecanoic acid	21.55	*p*-cresol	6.22	benzene, 1-ethoxy-4-methyl-	2.86
10	hexadecanoic acid, methyl ester	1.72	*E*,*E*,*Z*-1,3,12-nonadecatriene-5,14-diol	4.14	cyclohexanone, 3-ethenyl-	2.35	cyclohexanone, 3-ethenyl-	2
11	*n*-hexadecanoic acid	22.39	*n*-octadeca-6,9,12,15-tetraenoylpyrrolidide	8.2	1-hexadecanol	2.5	1-hexadecanol	2.68
12	9-octadecenoic acid, methyl ester, (*E*)-	4.55	oleic acid	10.43	*n*-hexadecanoic acid	21.5	*n*-hexadecanoic acid	21.37
13	9-octadecenoic acid, (*E*)-	3.81	2-propenoic acid, 3-(4-methoxyphenyl)-, 2-ethylhexyl ester	3.43	9,12-octadecadienoic acid (*Z*,*Z*)-, methyl ester	1.91	9-octadecenoic acid, methyl ester, (*E*)-	2.27
14	acetanilide	7.76	octadecanoic acid	12.77	9-octadecenoic acid, methyl ester, (E)-	6.97	oleic acid	4.73
15	oleic acid	7.88	octadecanamide	4.36	3,7,11,15-tetramethyl-2-hexadecen-1-ol	3.96	3-oxatricyclo[3.2.2.0(2,4)]nonane	10.11
16	octadecanoic acid	9.84	cyclononasiloxane, octadecamethyl-	2.62	7-hexadecenal, (*Z*)-	7.13	9-octadecenoic acid, (*E*)-	11.68
17	hexadecanamide	5.52	cyclononasiloxane, octadecamethyl-	2.35	9-octadecenoic acid, (*E*)-	11.76	2-propenoic acid, 3-(4-methoxyphenyl)-, 2-ethylhexyl ester	2.62
18	9-octadecenamide, (*Z*)-	1.67	bis(2-ethylhexyl) phthalate	1.9	octadecanoic acid	10.18	octadecanoic acid	10.97
19	cholest-4-ene	1.6	cyclononasiloxane, octadecamethyl-	2.23	hexadecanamide	1.74	hexadecanamide	5.2
20	cholesterol	2.61	cholesterol	3.2	9-octadecenamide, (*Z*)-	1.65	cholest-4-ene	1.79

In SS, lipids are crucial for producing long-chain fatty acids and esters during HTL, essential for biofuel’s carbon alkanes. These lipids, mainly triglycerides,^4^ break down into fatty acids and glycerol in HTL. Additionally, these fatty acids can undergo various changes, such as combining with amines under the amination reaction to form fatty acid amides such as hexadecanamide and octadecenamide (Table 2), transforming into alkanes and alkenes under the decarboxylation reaction, and reacting with amino acids to form N&O heterocyclic compounds.^35,36^

Proteins, another major component of SS, significantly impact the output of HTL. The formation of cyclic amides is the result of different reactions, including protein decarboxylation, deamination, dehydration, depolymerization, degradation, and self-condensation.^37^ It has also been discovered that amides can be formed via the acylation of fatty acids and amines.^38^ Moreover, the Maillard reaction between polysaccharides from carbohydrates and amino acids from proteins results in the formation of N-heterocyclic compounds such as pyrimidine-2-methyl.^39^ Simultaneously, amino acids undergo diamino-decarboxylation, generating hydrocarbons, and select oxygenated compounds, which migrate to the oil phase and enhance biocrude yields.

### 3.4 Role of Extraction Solvents for Nitrogen Concentration in Biocrude

To evaluate the effectiveness of the HTL in processing SS for biocrude production, a crucial aspect to consider is the N concentration in the resulting biocrudes. The N content is a key parameter affecting both the quality of the biocrude and its suitability for further applications. Consequently, this research contrasts the N concentrations obtained in this study with those reported in the existing literature focusing on HTL biocrudes of SS, specifically considering various extraction solvents. This comparison is instrumental in underscoring the distinctiveness and relevance of these study findings, illustrating how these results differ from or align with prior studies.

Table 3 presents a comprehensive summary of published literature concerning the HTL of SS. This table specifically details the N content in SS, outlines the HTL optimum conditions for the highest biocrudes used in various studies, and describes the extraction solvents employed for biocrudes. Additionally, it includes the reported N contents in the extracted biocrudes.

**Table 3:** List of Recent Researche on HTL of SS, Extracted Solvents, and Associated Results

feedstock	HTL optimum condition						HTL biocrude		ref
	N (wt %)	temperature (°C)	time (min)	pressure (MPa)	catalyst (% of SS)	biocrude extraction Solvent	yields (wt %)	N (wt %)	
SS		350	60	1.38	5.5% Red mud	DCM	38	4.4	(20)
						hexane	12	4.2	
						toluene	18	4.6	
						acetone	10	5.7	
secondary SS	5.81	340	10	0.2		DCM	22.9	4.88	(23)
secondary SS	7.37	350	15			acetone and diethyl ether	44.46	5.18	(40)
					2.5% K_2_CO_3_	acetone and diethyl ether	45	5	
SS	5.04	324	30			cyclohexane and acetone	27	1.40	(41)
					10% KF 851^a^		24	1.43	
					10% KF 1022-		21	3.69	
					10% ACF1600+		23	2.70	
SS	7.1	350	28.7	20	0.1 M Na_2_CO_3_	DCM	35.5	5	(42)
SS	4.60	350	15			DCM	19.37	6.53	(43)
					5% K_2_CO_3_		27.27	6.39	
					10% Al_2_O_3_		27.24	6.41	
					10% ATP∼		26.24	6.64	
					10% Co/Al_2_O_3_		28.31	6.53	
					5% Co/ATP		29.03	6.46	
					10% Co–Mo/Al_2_O_3_		28.93	6.53	
					10% Co–Mo/ATP		31.36	6.08	
SS	5.89	350	60			DCM	49.7	3.96	this study
						hexane	18.1	0.23	
						EB	50.1	0.32	
						EA	23.5	0.23	

a Nickel/molybdenum on activated alumina, -cobalt/molybdenum on activated alumina, + activated carbon felt, ∼Attapulgite.

Table 3 illustrates the performance of various solvents in extracting biocrude from the HTL of SS, highlighting significant differences in their extraction efficiencies. These variations are largely due to the distinct properties of each solvent, such as polarity and extraction capability. Notably, DCM consistently achieves higher extraction rates, with yields of 38,^20^ 35.5,^42^ and 49.7 wt % in this study, echoing findings from other research. This superior performance is potentially influenced by the varying organic content in SS, which differs based on location. For instance, SS’s organic content is reported to range from 30 to 50% in China,^44^ 40 to 50% in Europe,^45,46^ approximately 50% in the USA,^47^ and 60 to 80% in Japan.^48^

DCM, characterized by its polarity with a Polarity Index of 3.1, excels at extracting components like fatty acids, ester derivatives, and various oxygenates, both cyclic and noncyclic.^24^ However, studies have shown that SS is predominantly composed of nonpolar compounds,^28^ which presents a contrast to DCM’s extraction capabilities. Conversely, nonpolar solvents such as hexane and toluene yield less favorable extraction results as a consequence of their solvent properties. Notably, a synergistic effect is observed when combining polar and nonpolar solvents, exemplified by acetone (polar, with a PI of 5.1) and diethyl ether (nonpolar), leading to a significant increase in biocrude extraction efficiency, reaching 44.56 wt %.^40^ This outcome highlights the intricate nature of SS, comprising a mix of polar and nonpolar organic substances, further corroborated by similar findings with the cyclohexane and acetone combination.^41^

Additionally, the study references investigations on various catalysts, as detailed in Table 3, which focus on improving biocrude yields. However, these findings are not extensively discussed here, as the study’s primary objective is to evaluate the impact of different extraction solvents on both yields and N contents in biocrude. Polar solvents are effective in extracting polar organic compounds, and this proficiency extends to heteroatoms like N, which also display polar characteristics in biocrude. This capability of polar solvents significantly influences the composition of the extracted biocrude, particularly in terms of its N content. According to the data in Table 3, biocrude extracted with polar solvents exhibited higher nitrogen levels compared to those obtained using nonpolar solvents. Interestingly, biocrudes derived from a mix of polar and nonpolar solvents showed not only increased yields but also significantly lower nitrogen contents, with a notable reduction to 1.4 wt %.^41^

In this study, green solvents such as EB and EA demonstrated effective performance in terms of N content in biocrude extraction. Notably, EB showed remarkable potential, achieving a biocrude yield of 50.1 wt %, comparable to DCM at 49.7 wt %. EB, an ester, is formed by the combination of ethanol, alcohol, and butyric acid. The structure of esters includes a polar functional group, the carbonyl group present in the ester bond, as well as a nonpolar hydrocarbon chain. This dual structure imparts esters with a level of polarity, though generally, they are less polar than their constituent alcohols or acids and behave more like nonpolar.^49^ This attribute of EB, leaning toward a more nonpolar nature, is likely why it yielded biocrude with a minimal N content of just 0.23 wt %.

Given EB’s effectiveness as a green solvent, its high extraction efficiency, and its ability to yield biocrude with low N content, it stands out as a potential alternative to conventional organic solvents like DCM, which are more problematic in terms of environmental impact, sustainability, and the quality of biocrude. Moreover, biocrude with a lower heteroatom content, such as that extracted using EB, can be more readily upgraded to biofuels as it requires less processing compared to biocrude with higher heteroatom levels.

### 3.5 Nitrogen Distribution in All HTL Products

**Figure 5:**
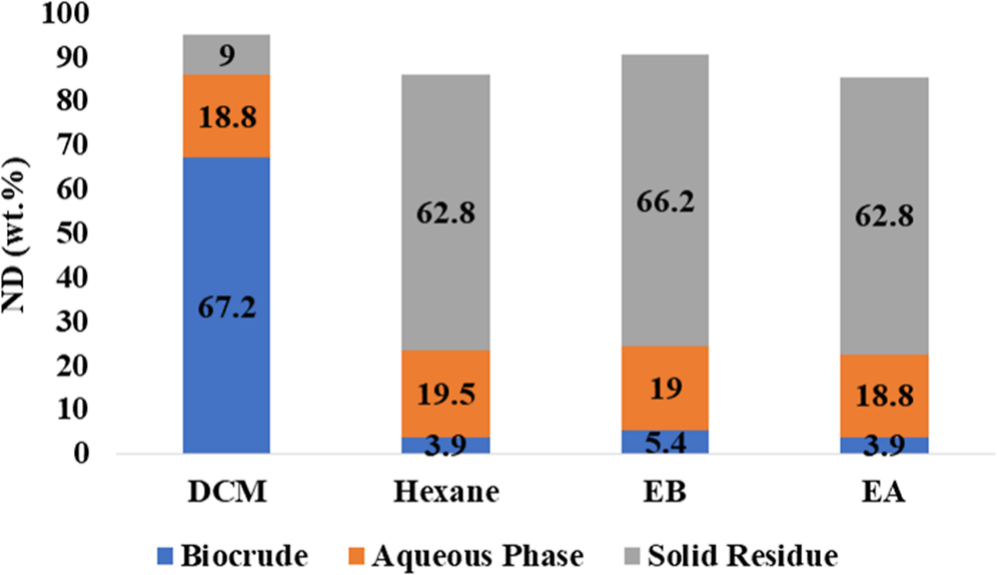
Nitrogen distribution in HTL products of SS.

In Figure 5, a comprehensive examination of the ND is presented across HTL products. The scrutiny of ND is confined to the biocrude, solid residue, and aqueous phases, with deliberate exclusion of the gas phase, given that it was not recovered and calculated by difference. It is imperative to note that the N balance, while a crucial metric, does not sum up to 100%. This discrepancy is a consequence of intentional exclusions, namely gas products, and inherent uncertainties in the experimental framework. The observed loss in recovery is attributed to multifaceted factors such as the volatilization of compounds during solvent removal, and the presence of experimental errors.

The biocrude extracted with DCM exhibited the highest N distribution at 67.23%, while all other biocrudes showed almost negligible levels, and a potential explanation for this disparity is elucidated in the “Elemental Composition of HTL Biocrudes” section above. On the other hand, the N distribution remains remarkably consistent across all aqueous phases, hovering around 18 to 19 wt %. This observed similarity is likely attributed to the consistent operating conditions maintained throughout the experiments, indicating robust and stable performance across all aqueous phases. The N content in the solid residues varied significantly among the different solvents used. Notably, DCM exhibited the lowest nitrogen content in its solid residue, a consequence of its superior ability to extract N and S compounds, as detailed in the section “Elemental Composition of HTL Biocrudes”. In contrast, residues from other solvents showed higher N levels, likely due to their lower extraction efficiencies, resulting in N-containing compounds remaining in the solid residues. Furthermore, the HHVs of the solid residues were observed to be greater than those of the initial feedstock. This increase can be attributed to the removal of low-energy volatiles through processes such as hydrolysis, dehydration, and decarboxylation, coupled with a reduction in the initial mass.

### 3.6 Energy Recovery

**Figure 6:**
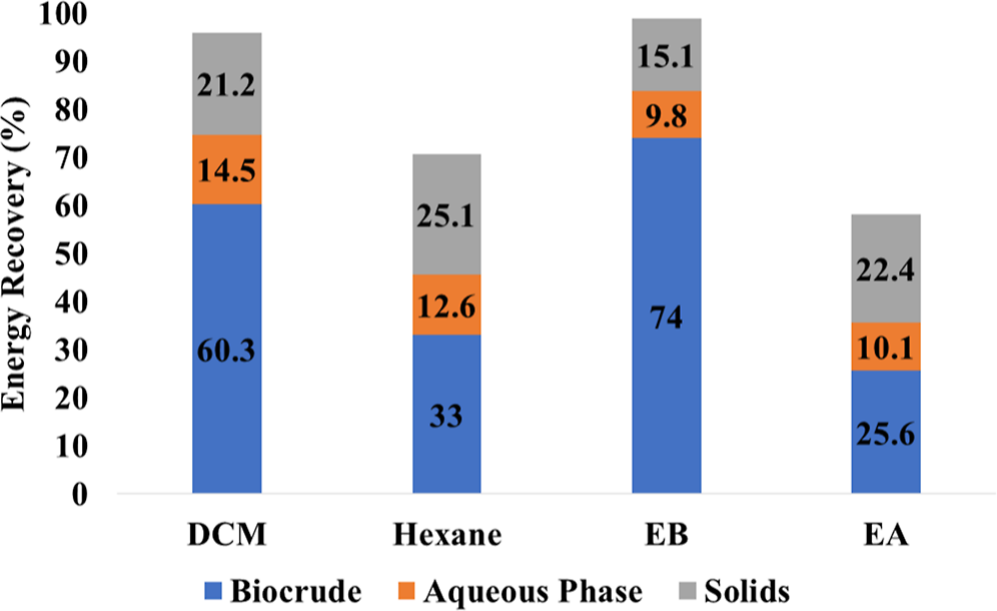
Energy recovery in HTL products of SS.

Efficiency in the HTL process relies on recovering hydrocarbon species (CHNS) and energy from the feedstock to biocrude. Optimal feedstocks for maximal carbon and energy recovery are recommended. Limited data exist on CHNS and energy recovery in various HTL products (biocrude, solid residues, and aqueous) from SS. Understanding hydrocarbon species and energy recovery (ER), especially in SS HTL biocrude, is vital for optimizing conditions and improving large-scale biocrude production efficiency. The CHNS results of HTL biocrude have been reported in Table 1, and the ER of HTL products is shown in Figure 6.

EB-extracted biocrude not only demonstrated the highest ER at 74%, surpassing DCM (60.2%), hexane (33%), and EA (25.6%), but also exhibited the highest extraction yield. This performance aligns with biocrude extraction yields and CHNS concentrations (Table 1). Other products maintained a consistent ER with minor variations. These findings underscore SS as a promising candidate for HTL conversion into potential biocrude, with EB emerging as a sustainable option for the highest extraction yield, highlighting its green solvent attributes for enhanced sustainability and efficiency.

### 3.7 Limitations of the Study

GC–MS analysis was utilized to characterize the biocrude from SS via HTL. However, it is important to note limitations such as the finite capacity of GC columns leading to peak overlap and poor resolution, potential bias toward volatile compounds, matrix effects interfering with compound detection, and limited representation of high molecular weight compounds. While GC–MS provided valuable insights into the volatile fraction of the biocrude, these limitations should be considered, and future studies may benefit from complementary analytical techniques for a more comprehensive analysis.

## 4 Conclusions and Future Prospects

This study highlights the HTL for SS, emphasizing the pivotal role of solvent choice in efficient and sustainable biocrude extraction. EB, a green alternative, matches conventional solvents in extraction yield while achieving remarkably low nitrogen contents and high ER, suggesting potential reductions in energy and purification costs. Unveiling overlooked byproducts, the aqueous phase, and solid residues showcase high ND, hinting at untapped applications. The adoption of green solvents, like EB, signals a crucial step toward eco-friendly SS management, aligning with global sustainability goals.

Considering the scalability of this process, future research should prioritize efficient solvent recovery methods to minimize waste and enhance economic and environmental sustainability. This includes investigating solvent–solute interactions for optimized usage. Furthermore, exploring solvent recycling strategies and assessing large-scale economic viability are crucial. Ultimately, this study’s findings not only demonstrate high extraction efficiency, energy recovery, and biocrude quality but also pave the way for sustainable waste-to-energy technologies.
